# Human Metapneumovirus and Respiratory Syncytial Virus, Brazil

**DOI:** 10.3201/eid0912.030522

**Published:** 2003-12

**Authors:** Luis E. Cuevas, Abubaker M. Ben Nasser, Winifred Dove, Ricardo Q. Gurgel, Julie Greensill, C. Anthony Hart

**Affiliations:** * Liverpool School of Tropical Medicine, Liverpool, United Kingdom; † University of Liverpool, Liverpool, United Kingdom; ‡ Federal University of Sergipe, Aracaju, Brazil

**Keywords:** Human metapneumovirus, respiratory syncytial virus, acute respiratory infections, children, epidemiology, Brazil

## Abstract

We describe the epidemiologic and clinical characteristics of 111 children attending clinics and hospitals in Aracaju, northeast Brazil, with acute respiratory infections attributable to human metapneumovirus (HMPV), respiratory syncytial virus (RSV), or both in May and June 2002. Fifty-three (48%) children were infected with RSV alone, 19 (17%) with HMPV alone, and 8 (7%) had RSV/HMPV co-infections.

Human metapneumovirus (HMPV) was first identified in the Netherlands in 2001 (*1*) and was implicated as a potential etiologic agent for respiratory infections. Since then, the virus has been reported from other European countries (*2*–*6*), Asia (*7*–*9*), and North America (*10*–*13*), findings that suggest it has a worldwide distribution. However, HMPV has not been reported from South America. We describe the epidemiologic and clinical characteristics of 111 children attending clinics and hospitals in Aracaju, northeast Brazil, with acute respiratory infections attributable to HMPV, respiratory syncytial virus (RSV), or both.

## The Study

Children <3 years of age attending two health centers, one public reference hospital (Joao Alves), and a private hospital in Aracaju were invited to participate in our study during April and May 2002. These months correspond to the beginning of the rainy season, when most cases of bronchiolitis occur. The health centers provide 24-hour medical services for acute illnesses; patients with severe health problems are referred to the public hospital. The small private hospital caters to self-referred and transferred patients (mostly from the public hospital). Children with diagnoses of acute lower respiratory infections (ALRI) were recruited consecutively after informed parental consent. The diagnosis of ALRI was based on the presence of cough, tachypnea, chest indrawing, or wheeze of <7 days’ duration and followed the World Health Organization’s standard protocol for research on ALRI (*14*).

Nasopharyngeal secretions were collected by using sterile mucous traps (Maesk Medical A/S, Bettina Bay, Denmark). Immediately after collection, aspirates were mixed with phosphate-buffered saline, transferred into 2-mL cryotubes, and kept frozen at –80°C until analyzed. Hypoxia levels were measured by pulse oximetry before the use of oxygen, and children were treated according to local guidelines for the management of ALRI.

Detection of the HMPV genome was performed by reverse transcription–polymerase chain reaction amplification (RT-PCR) of the matrix (M), fusion (F) and nucleocapsid (N) protein genes, as described previously (*5*). Selected PCR products were cloned into a TA cloning vector (pGEM-T, Promega, UK), and the sequence was determined to confirm the identity of the virus detected by the PCR reaction. Samples were defined as HMPV positive if at least two of the RT-PCR test results were positive, although in practice all positive samples had at least two positive PCRs. Detection of RSV by RT-PCR was as described by Fletcher et al. (*15*).

A total of 111 children (57 from the health centers, 25 from Joao Alvez Hospital, and 29 from the private hospital) were recruited. Their ages ranged from 1 to 30 months (median 7 months). Fifty-three (48%) children were infected with RSV alone, 19 (17%) with HMPV alone, and 8 (7%) with both (co-infections). Forty-six (88%) of the RSV cases were of group A and 6 (12%) group B.

**Figure 1:**
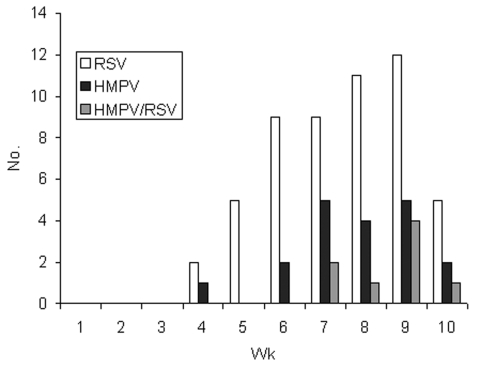
Number of children with respiratory syncytial virus (RSV), human metapneumovirus (HMPV), and RSV/HMPV co-infection by study week (week 10 incomplete).

The incidence of ALRI increased during the 10 weeks of the study from no cases in the first 2 weeks, 4 cases in the next 2 weeks, and up to 25 cases in week 9 (Figure 1). RSV and HMPV infections appeared to coincide in time during the period of the study.

**Figure 2:**
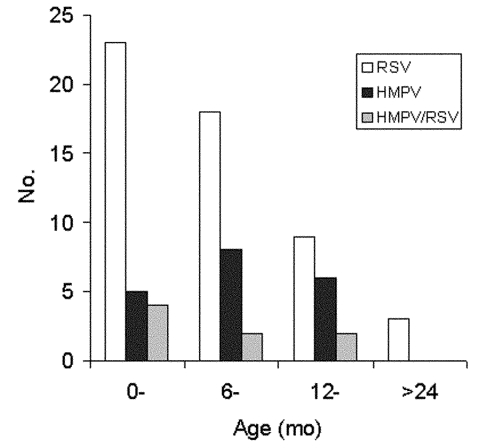
Number of children with respiratory syncytial virus (RSV), human metapneumovirus (HMPV), and RSV/HMPV co-infection by age.

The characteristics of the children are described in the Table. The incidence of HMPV and RSV varied according to the enrollment site. Eighteen (32%) of the 57 patients attending the health centers had HMPV compared to 1 (4%) of the 25 infants attending the reference hospital (p = 0.01). In contrast, RSV was significantly (p = 0.01) more frequently detected in patients attending the referral hospital (17 [68%] of 25) than the health centers (23 [40%] of 57). The age distribution of the infected infants is shown in Figure 2. The greatest number of cases of RSV was observed in children <12 months of age. HMPV infection appeared to be more frequent in children 6–24 months of age, although this finding was not statistically significant.

**Table:** Characteristics of children with human metapneumovirus (HMPV), respiratory syncytial virus (RSV), and RSV/HMPV co-infections^a^

Characteristics	HMPV	RSV	RSV/HMPV co-infection	HMPV/RSV negative	
	n = 19	n = 53	n = 8	n = 31	
Age in mo, mean (SD)	10.1 (5.4)	8.9 (7.2)	8.2 (6.0)	9.8 (7.7)	
Recruitment site^b^					
Health center	12 (21)	23 (40.3)	6 (10.5)	16 (28.1)	
Private hospital	6 (20.7)	13 (44.8)	2 (6.9)	8 (27.6)	
Public hospital	1 (4)	17 (68)	0 (0)	7 (28)	
Male:female (% male)	12:7 (63)	36:17 (68)	4:4 (50)	21:10 (68)	
Birthweight <2,500 g	4 (21)	2 (4)	0 (0)	2 (6.5)	
Previous admission	2 (10)	7 (13)	3 (38)	1 (3)	
History of allergies	2 (11)	6 (11)	1 (13)	4 (13)	
Clinical signs and symptoms					
Respiratory rate, mean (SD)	44 (10.1)	51 (12.3)^c^	54 (16.2)	46 (10.1)	
Temperature >37.5°C	15 (79)	43 (81)	8 (100)	23 (74)	
Runny nose	18 (95)	45 (85)	6 (75)	26 (84)	
Cyanosis	0 (0)	4 (7.5)	0 (0)	2 (6.5)	
Cough	19 (100)	53 (100)	8 (100)	31 (100)	
Wheezing	9 (47.4)	31 (58.5)	2 (25%)	19 (61.3)	
Chest indrawings	4 (21)	19 (36)	3 (37.5)	8 (25.8)	
pO_2_ <90%	1 (5.3)	10 (18.9)	0 (0)	1 (3.2)	
pO_2_ <94%^c^	5 (26.3)	25 (47.2)	5 (62.5)	9 (29)	

^a^Figures are frequencies (%), unless otherwise specified. ^b^Health centers vs. public hospital, p < 0.01. Percentages are row percentages. ^c^p < 0.05.

All children had coughing; 61 (55%) were wheezing, which was audible without auscultation; and 34 (31%) had chest indrawing. The mean respiratory rate on consultation was higher in patients with RSV than in patients with HMPV infection (p = 0.03). Similarly, wheezing was more often audible in children infected with RSV (59%) than in children with HMPV (47%) or RSV/HMPV co-infections (25%), although this finding did not reach statistical significance (chi square for trend, p = 0.07). Eighteen (16%) children had severe hypoxia with pO_2_ ≤90%, and 44 (40%) had pO_2_ <94%. The presence of the viruses was associated with the degree of hypoxia. Five (26%) of the 19 children with HMPV had pO_2_ <94% compared to 25 (47%) of the 53 with RSV and 5 (63%) of the 8 with RSV/HMPV co-infection (chi square for trend, p = 0.05). Severe hypoxia (pO_2_ <90%), however, was less frequent in children with HMPV (1 [5%] of 19) or RSV/HMPV co-infections (1 [13%] of 8) than in children with RSV infection (14 [26%] of 53 (chi square for trend, p = 0.04). The lower prevalence of hypoxia suggests that children with HMPV had milder illnesses, since only 5 (26%) of the 19 HMPV patients and 2 (25%) of the 8 patients with RSV/HMPV co-infections were admitted to hospital compared with 27 (51%) of the 53 with RSV infection, although this finding was not statistically significant.

## Conclusions

This study is the first to describe HMPV in Latin America. To date, information on the clinical signs and symptoms and epidemiology of HMPV infection is limited. Our study indicates that HMPV and RSV may have similar signs and symptoms and can present as a co-infection.

The incidence of HMPV increased at the same time that the number of cases with RSV was increasing. HMPV, however, was more prevalent in children attending peripheral clinics than the referral hospital. Similarly, children with HMPV were less likely to be hypoxic and had lower respiratory rates than those with RSV, which suggests that HMPV infection may result in milder clinical signs and symptoms. Further studies, however, are necessary to explore if coinfected children are more or less likely to have a worse clinical outcome than children infected with only one virus. A recent report of hospitalized children in intensive care suggested that dual infections could be associated with increased severity (*5*). An alternative explanation to reconcile these findings is the possibility that different HMPV subgroups (*2*,*9*,*10*,*13*,*16*) produce clinical syndromes of varying severity.

Caution is necessary to interpret our findings as this was a cross-sectional study. Virus in nasopharyngeal secretions does not prove that it is the cause of the respiratory symptoms, as we did not investigate the prevalence of the virus in asymptomatic children or the natural history of the infection. HMPV, however, has been associated with community-acquired respiratory illnesses (*2*,*6*,*9*–*12*) and severe ALRI in immunocompromised patients (*17*,*18*), and the virus was likely responsible for the clinical illness in our children. Several studies have also described an association between HMPV and acute wheezing (*3*–*5*,*8*). Although wheezing was a feature in 47% of the HMPV-infected children, a greater proportion (59%) of children infected with RSV had wheezing. The association between RSV infection and asthma is well studied. However, most children are exposed to RSV infections before the age of 2 years, and a combination of factors is likely required (*19*). The coincidental timing of RSV and HMPV might have led us to miss the true cause. The role of HMPV in ALRI and asthma merits further investigation. Future studies should aim to establish the natural history and clinical outcome of these infections.
